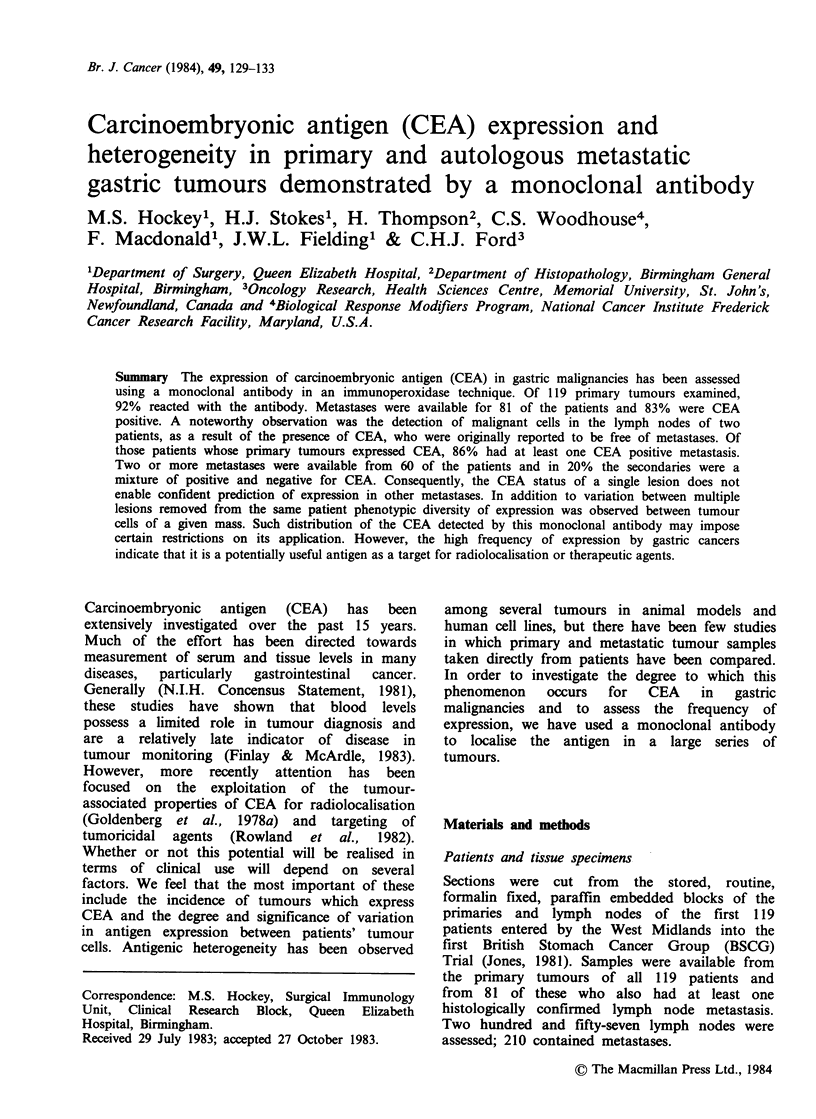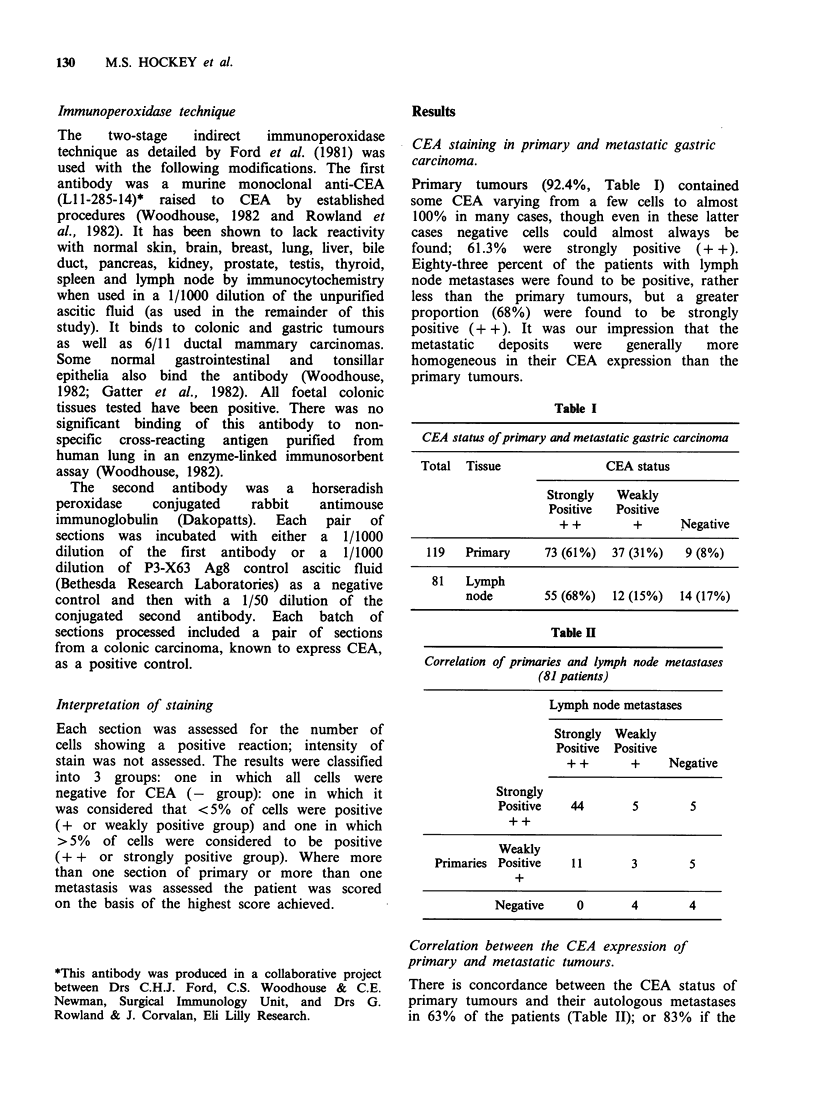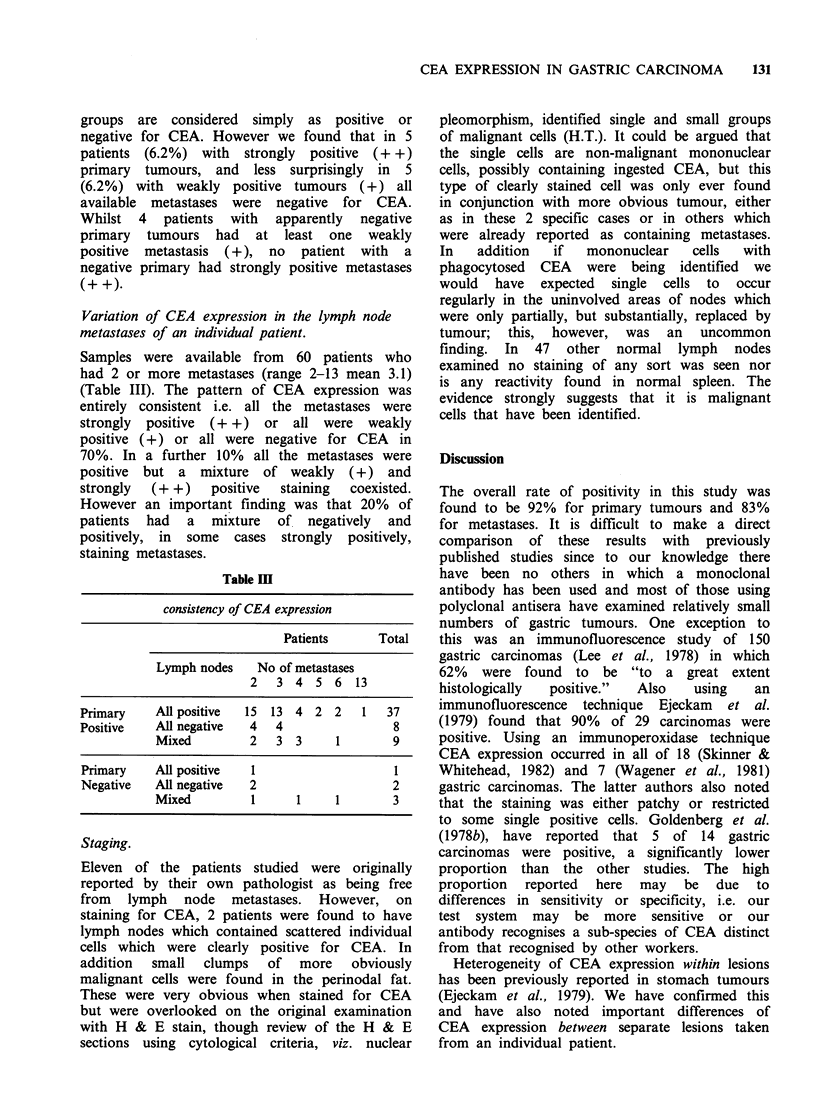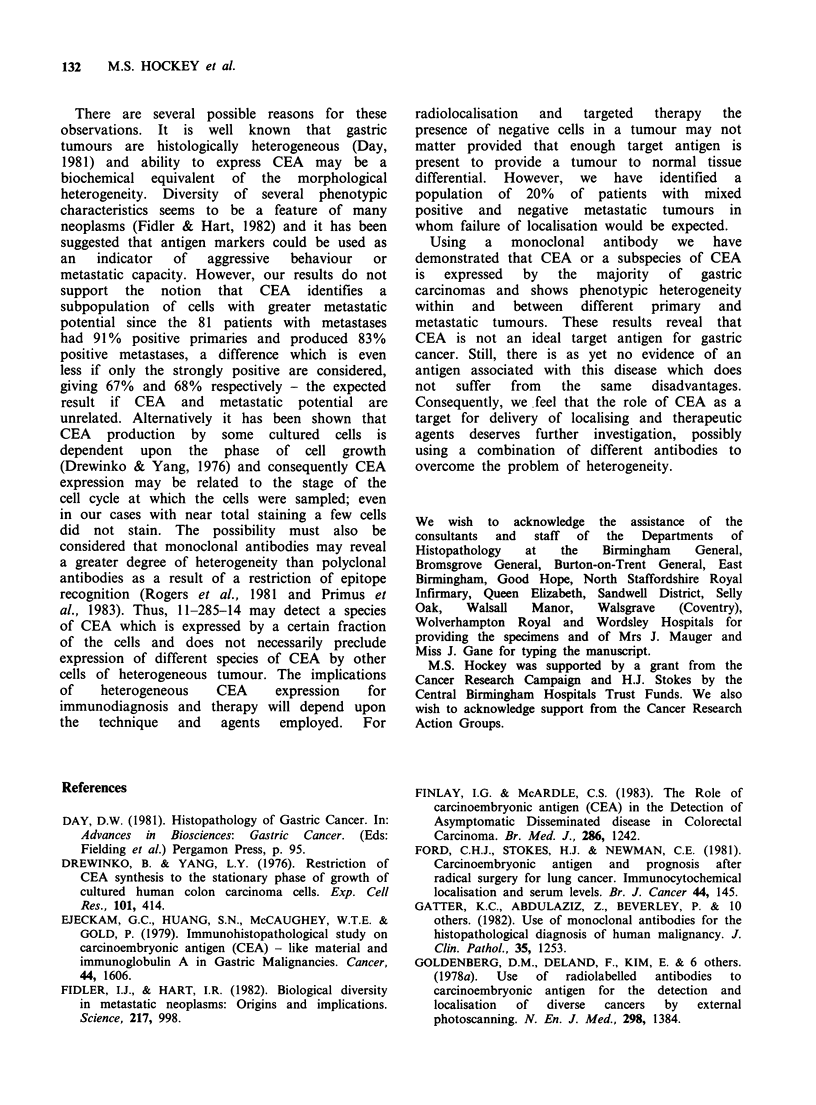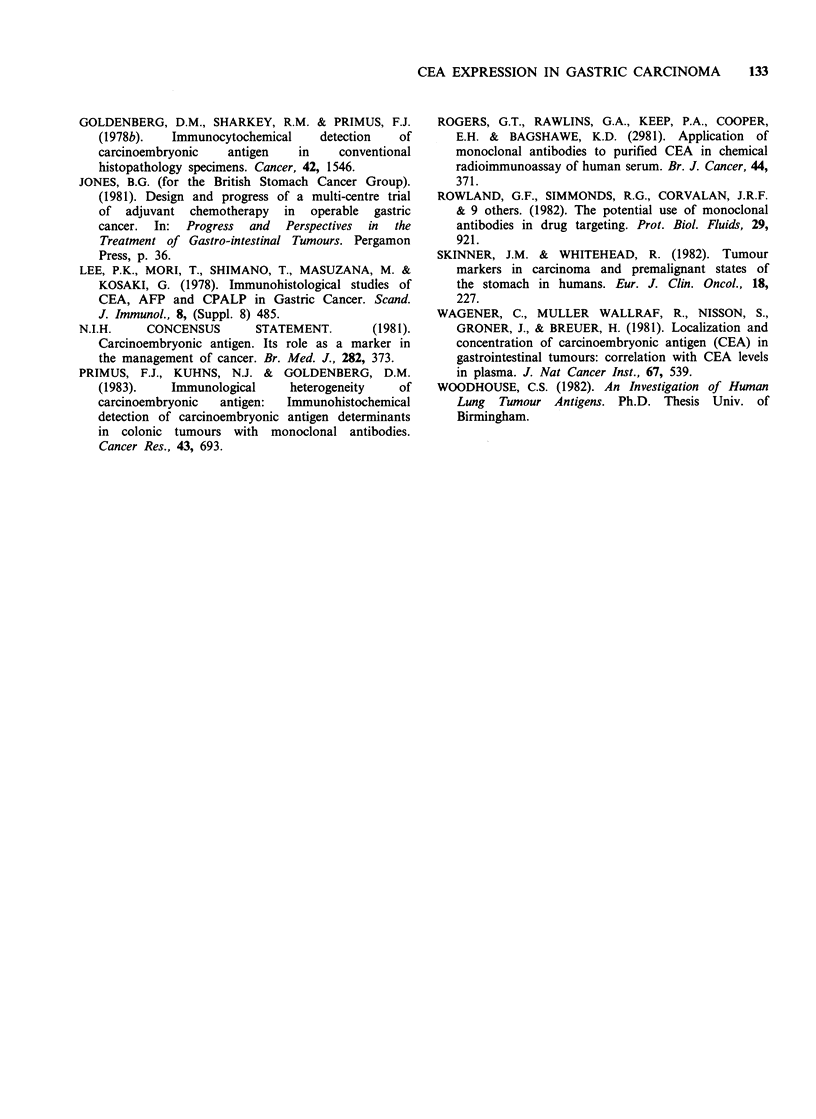# Carcinoembryonic antigen (CEA) expression and heterogeneity in primary and autologous metastatic gastric tumours demonstrated by a monoclonal antibody.

**DOI:** 10.1038/bjc.1984.24

**Published:** 1984-02

**Authors:** M. S. Hockey, H. J. Stokes, H. Thompson, C. S. Woodhouse, F. Macdonald, J. W. Fielding, C. H. Ford

## Abstract

The expression of carcinoembryonic antigen (CEA) in gastric malignancies has been assessed using a monoclonal antibody in an immunoperoxidase technique. Of 119 primary tumours examined, 92% reacted with the antibody. Metastases were available for 81 of the patients and 83% were CEA positive. A noteworthy observation was the detection of malignant cells in the lymph nodes of two patients, as a result of the presence of CEA, who were originally reported to be free of metastases. Of those patients whose primary tumours expressed CEA, 86% had at least one CEA positive metastasis. Two or more metastases were available from 60 of the patients and in 20% the secondaries were a mixture of positive and negative for CEA. Consequently, the CEA status of a single lesion does not enable confident prediction of expression in other metastases. In addition to variation between multiple lesions removed from the same patient phenotypic diversity of expression was observed between tumour cells of a given mass. Such distribution of the CEA detected by this monoclonal antibody may impose certain restrictions on its application. However, the high frequency of expression by gastric cancers indicate that it is a potentially useful antigen as a target for radiolocalisation or therapeutic agents.


Br. J. Cancer (1984), 49, 129–133

### Carcinoembryonic antigen (CEA) expression and heterogeneity in primary and autologous metastatic gastric tumours demonstrated by a monoclonal antibody

M.S. Hockey[1], H.J. Stokes[1], H. Thompson[2], C.S. Woodhouse[4], F. Macdonald[1], J.W.L. Fielding[1] & C.H.J. Ford[3]

[1]*Department of Surgery, Queen Elizabeth Hospital,* [2]*Department of Histopathology, Birmingham General Hospital, Birmingham,* [3]*Oncology Research, Health Sciences Centre, Memorial University, St. John's, Newfoundland, Canada and* [4]*Biological Response Modifiers Program, National Cancer Institute Frederick Cancer Research Facility, Maryland, U.S.A.*

**Summary** The expression of carcinoembryonic antigen (CEA) in gastric malignancies has been assessed using a monoclonal antibody in an immunoperoxidase technique. Of 119 primary tumours examined, 92% reacted with the antibody. Metastases were available for 81 of the patients and 83% were CEA positive. A noteworthy observation was the detection of malignant cells in the lymph nodes of two patients, as a result of the presence of CEA, who were originally reported to be free of metastases. Of those patients whose primary tumours expressed CEA, 86% had at least one CEA positive metastasis. Two or more metastases were available from 60 of the patients and in 20% the secondaries were a mixture of positive and negative for CEA. Consequently, the CEA status of a single lesion does not enable confident prediction of expression in other metastases. In addition to variation between multiple lesions removed from the same patient phenotypic diversity of expression was observed between tumour cells of a given mass. Such distribution of the CEA detected by this monoclonal antibody may impose certain restrictions on its application. However, the high frequency of expression by gastric cancers indicate that it is a potentially useful antigen as a target for radiolocalisation or therapeutic agents.

Carcinoembryonic antigen (CEA) has been extensively investigated over the past 15 years. Much of the effort has been directed towards measurement of serum and tissue levels in many diseases, particularly gastrointestinal cancer. Generally (N.I.H. Concensus Statement, 1981), these studies have shown that blood levels possess a limited role in tumour diagnosis and are a relatively late indicator of disease in tumour monitoring (Finlay & McArdle, 1983). However, more recently attention has been focused on the exploitation of the tumour-associated properties of CEA for radiolocalisation (Goldenberg *et al.*, 1978*a*) and targeting of tumoricidal agents (Rowland *et al.*, 1982). Whether or not this potential will be realised in terms of clinical use will depend on several factors. We feel that the most important of these include the incidence of tumours which express CEA and the degree and significance of variation in antigen expression between patients' tumour cells. Antigenic heterogeneity has been observed among several tumours in animal models and human cell lines, but there have been few studies in which primary and metastatic tumour samples taken directly from patients have been compared. In order to investigate the degree to which this phenomenon occurs for CEA in gastric malignancies and to assess the frequency of expression, we have used a monoclonal antibody to localise the antigen in a large series of tumours.

Correspondence: M.S. Hockey, Surgical Immunology Unit, Clinical Research Block, Queen Elizabeth Hospital, Birmingham.
Received 29 July 1983; accepted 27 October 1983.

### Materials and methods

### *Patients and tissue specimens*

Sections were cut from the stored, routine, formalin fixed, paraffin embedded blocks of the primaries and lymph nodes of the first 119 patients entered by the West Midlands into the first British Stomach Cancer Group (BSCG) Trial (Jones, 1981). Samples were available from the primary tumours of all 119 patients and from 81 of these who also had at least one histologically confirmed lymph node metastasis. Two hundred and fifty-seven lymph nodes were assessed; 210 contained metastases.

© The Macmillan Press Ltd., 1984

*Immunoperoxidase technique*

The two-stage indirect immunoperoxidase technique as detailed by Ford *et al.* (1981) was used with the following modifications. The first antibody was a murine monoclonal anti-CEA (L11-285-14)* raised to CEA by established procedures (Woodhouse, 1982 and Rowland *et al.*, 1982). It has been shown to lack reactivity with normal skin, brain, breast, lung, liver, bile duct, pancreas, kidney, prostate, testis, thyroid, spleen and lymph node by immunocytochemistry when used in a 1/1000 dilution of the unpurified ascitic fluid (as used in the remainder of this study). It binds to colonic and gastric tumours as well as 6/11 ductal mammary carcinomas. Some normal gastrointestinal and tonsillar epithelia also bind the antibody (Woodhouse, 1982; Gatter *et al.*, 1982). All foetal colonic tissues tested have been positive. There was no significant binding of this antibody to non-specific cross-reacting antigen purified from human lung in an enzyme-linked immunosorbent assay (Woodhouse, 1982).

The second antibody was a horseradish peroxidase conjugated rabbit antimouse immunoglobulin (Dakopatts). Each pair of sections was incubated with either a 1/1000 dilution of the first antibody or a 1/1000 dilution of P3-X63 Ag8 control ascitic fluid (Bethesda Research Laboratories) as a negative control and then with a 1/50 dilution of the conjugated second antibody. Each batch of sections processed included a pair of sections from a colonic carcinoma, known to express CEA, as a positive control.

*Interpretation of staining*

Each section was assessed for the number of cells showing a positive reaction; intensity of stain was not assessed. The results were classified into 3 groups: one in which all cells were negative for CEA (− group): one in which it was considered that <5% of cells were positive (+ or weakly positive group) and one in which >5% of cells were considered to be positive (++ or strongly positive group). Where more than one section of primary or more than one metastasis was assessed the patient was scored on the basis of the highest score achieved.

*This antibody was produced in a collaborative project between Drs C.H.J. Ford, C.S. Woodhouse & C.E. Newman, Surgical Immunology Unit, and Drs G. Rowland & J. Corvalan, Eli Lilly Research.

### Results

*CEA staining in primary and metastatic gastric carcinoma.*

Primary tumours (92.4%, Table I) contained some CEA varying from a few cells to almost 100% in many cases, though even in these latter cases negative cells could almost always be found; 61.3% were strongly positive (++). Eighty-three percent of the patients with lymph node metastases were found to be positive, rather less than the primary tumours, but a greater proportion (68%) were found to be strongly positive (++). It was our impression that the metastatic deposits were generally more homogeneous in their CEA expression than the primary tumours.

**Table I**

*CEA status of primary and metastatic gastric carcinoma*

| Total | Tissue | CEA status | | |
|---|---|---|---|---|
| | | Strongly Positive ++ | Weakly Positive + | Negative |
| 119 | Primary | 73 (61%) | 37 (31%) | 9 (8%) |
| 81 | Lymph node | 55 (68%) | 12 (15%) | 14 (17%) |

**Table II**

*Correlation of primaries and lymph node metastases (81 patients)*

| | | Lymph node metastases | | |
|---|---|---|---|---|
| | | Strongly Positive ++ | Weakly Positive + | Negative |
| | Strongly Positive ++ | 44 | 5 | 5 |
| Primaries | Weakly Positive + | 11 | 3 | 5 |
| | Negative | 0 | 4 | 4 |

*Correlation between the CEA expression of primary and metastatic tumours.*

There is concordance between the CEA status of primary tumours and their autologous metastases in 63% of the patients (Table II); or 83% if the groups are considered simply as positive or negative for CEA. However we found that in 5 patients (6.2%) with strongly positive (++) primary tumours, and less surprisingly in 5 (6.2%) with weakly positive tumours (+) all available metastases were negative for CEA. Whilst 4 patients with apparently negative primary tumours had at least one weakly positive metastasis (+), no patient with a negative primary had strongly positive metastases (++).

*Variation of CEA expression in the lymph node metastases of an individual patient.*

Samples were available from 60 patients who had 2 or more metastases (range 2–13 mean 3.1) (Table III). The pattern of CEA expression was entirely consistent i.e. all the metastases were strongly positive (++) or all were weakly positive (+) or all were negative for CEA in 70%. In a further 10% all the metastases were positive but a mixture of weakly (+) and strongly (++) positive staining coexisted. However an important finding was that 20% of patients had a mixture of negatively and positively, in some cases strongly positively, staining metastases.

**Table III**

*consistency of CEA expression*

| | | Patients | | | | | | Total |
|---|---|---|---|---|---|---|---|---|
| | Lymph nodes | No of metastases | | | | | | |
| | | 2 | 3 | 4 | 5 | 6 | 13 | |
| Primary Positive | All positive | 15 | 13 | 4 | 2 | 2 | 1 | 37 |
| | All negative | 4 | 4 | | | | | 8 |
| | Mixed | 2 | 3 | 3 | | 1 | | 9 |
| Primary Negative | All positive | 1 | | | | | | 1 |
| | All negative | 2 | | | | | | 2 |
| | Mixed | 1 | | 1 | | 1 | | 3 |

*Staging.*

Eleven of the patients studied were originally reported by their own pathologist as being free from lymph node metastases. However, on staining for CEA, 2 patients were found to have lymph nodes which contained scattered individual cells which were clearly positive for CEA. In addition small clumps of more obviously malignant cells were found in the perinodal fat. These were very obvious when stained for CEA but were overlooked on the original examination with H & E stain, though review of the H & E sections using cytological criteria, *viz.* nuclear pleomorphism, identified single and small groups of malignant cells (H.T.). It could be argued that the single cells are non-malignant mononuclear cells, possibly containing ingested CEA, but this type of clearly stained cell was only ever found in conjunction with more obvious tumour, either as in these 2 specific cases or in others which were already reported as containing metastases. In addition if mononuclear cells with phagocytosed CEA were being identified we would have expected single cells to occur regularly in the uninvolved areas of nodes which were only partially, but substantially, replaced by tumour; this, however, was an uncommon finding. In 47 other normal lymph nodes examined no staining of any sort was seen nor is any reactivity found in normal spleen. The evidence strongly suggests that it is malignant cells that have been identified.

### Discussion

The overall rate of positivity in this study was found to be 92% for primary tumours and 83% for metastases. It is difficult to make a direct comparison of these results with previously published studies since to our knowledge there have been no others in which a monoclonal antibody has been used and most of those using polyclonal antisera have examined relatively small numbers of gastric tumours. One exception to this was an immunofluorescence study of 150 gastric carcinomas (Lee *et al.*, 1978) in which 62% were found to be "to a great extent histologically positive." Also using an immunofluorescence technique Ejeckam *et al.* (1979) found that 90% of 29 carcinomas were positive. Using an immunoperoxidase technique CEA expression occurred in all of 18 (Skinner & Whitehead, 1982) and 7 (Wagener *et al.*, 1981) gastric carcinomas. The latter authors also noted that the staining was either patchy or restricted to some single positive cells. Goldenberg *et al.* (1978*b*), have reported that 5 of 14 gastric carcinomas were positive, a significantly lower proportion than the other studies. The high proportion reported here may be due to differences in sensitivity or specificity, i.e. our test system may be more sensitive or our antibody recognises a sub-species of CEA distinct from that recognised by other workers.

Heterogeneity of CEA expression *within* lesions has been previously reported in stomach tumours (Ejeckam *et al.*, 1979). We have confirmed this and have also noted important differences of CEA expression *between* separate lesions taken from an individual patient.

There are several possible reasons for these observations. It is well known that gastric tumours are histologically heterogeneous (Day, 1981) and ability to express CEA may be a biochemical equivalent of the morphological heterogeneity. Diversity of several phenotypic characteristics seems to be a feature of many neoplasms (Fidler & Hart, 1982) and it has been suggested that antigen markers could be used as an indicator of aggressive behaviour or metastatic capacity. However, our results do not support the notion that CEA identifies a subpopulation of cells with greater metastatic potential since the 81 patients with metastases had 91% positive primaries and produced 83% positive metastases, a difference which is even less if only the strongly positive are considered, giving 67% and 68% respectively – the expected result if CEA and metastatic potential are unrelated. Alternatively it has been shown that CEA production by some cultured cells is dependent upon the phase of cell growth (Drewinko & Yang, 1976) and consequently CEA expression may be related to the stage of the cell cycle at which the cells were sampled; even in our cases with near total staining a few cells did not stain. The possibility must also be considered that monoclonal antibodies may reveal a greater degree of heterogeneity than polyclonal antibodies as a result of a restriction of epitope recognition (Rogers *et al.*, 1981 and Primus *et al.*, 1983). Thus, 11–285–14 may detect a species of CEA which is expressed by a certain fraction of the cells and does not necessarily preclude expression of different species of CEA by other cells of heterogeneous tumour. The implications of heterogeneous CEA expression for immunodiagnosis and therapy will depend upon the technique and agents employed. For radiolocalisation and targeted therapy the presence of negative cells in a tumour may not matter provided that enough target antigen is present to provide a tumour to normal tissue differential. However, we have identified a population of 20% of patients with mixed positive and negative metastatic tumours in whom failure of localisation would be expected.

Using a monoclonal antibody we have demonstrated that CEA or a subspecies of CEA is expressed by the majority of gastric carcinomas and shows phenotypic heterogeneity within and between different primary and metastatic tumours. These results reveal that CEA is not an ideal target antigen for gastric cancer. Still, there is as yet no evidence of an antigen associated with this disease which does not suffer from the same disadvantages. Consequently, we feel that the role of CEA as a target for delivery of localising and therapeutic agents deserves further investigation, possibly using a combination of different antibodies to overcome the problem of heterogeneity.

We wish to acknowledge the assistance of the consultants and staff of the Departments of Histopathology at the Birmingham General, Bromsgrove General, Burton-on-Trent General, East Birmingham, Good Hope, North Staffordshire Royal Infirmary, Queen Elizabeth, Sandwell District, Selly Oak, Walsall Manor, Walsgrave (Coventry), Wolverhampton Royal and Wordsley Hospitals for providing the specimens and of Mrs J. Mauger and Miss J. Gane for typing the manuscript.

M.S. Hockey was supported by a grant from the Cancer Research Campaign and H.J. Stokes by the Central Birmingham Hospitals Trust Funds. We also wish to acknowledge support from the Cancer Research Action Groups.

### References

DAY, D.W. (1981). Histopathology of Gastric Cancer. In: *Advances in Biosciences*: *Gastric Cancer*. (Eds: Fielding *et al.*) Pergamon Press, p. 95.

DREWINKO, B. & YANG, L.Y. (1976). Restriction of CEA synthesis to the stationary phase of growth of cultured human colon carcinoma cells. *Exp. Cell Res.*, **101**, 414.

EJECKAM, G.C., HUANG, S.N., McCAUGHEY, W.T.E. & GOLD, P. (1979). Immunohistopathological study on carcinoembryonic antigen (CEA) – like material and immunoglobulin A in Gastric Malignancies. *Cancer*, **44**, 1606.

FIDLER, I.J., & HART, I.R. (1982). Biological diversity in metastatic neoplasms: Origins and implications. *Science*, **217**, 998.

FINLAY, I.G. & McARDLE, C.S. (1983). The Role of carcinoembryonic antigen (CEA) in the Detection of Asymptomatic Disseminated disease in Colorectal Carcinoma. *Br. Med. J.*, **286**, 1242.

FORD, C.H.J., STOKES, H.J. & NEWMAN, C.E. (1981). Carcinoembryonic antigen and prognosis after radical surgery for lung cancer. Immunocytochemical localisation and serum levels. *Br. J. Cancer* **44**, 145.

GATTER, K.C., ABDULAZIZ, Z., BEVERLEY, P. & 10 others. (1982). Use of monoclonal antibodies for the histopathological diagnosis of human malignancy. *J. Clin. Pathol.*, **35**, 1253.

GOLDENBERG, D.M., DELAND, F., KIM, E. & 6 others. (1978*a*). Use of radiolabelled antibodies to carcinoembryonic antigen for the detection and localisation of diverse cancers by external photoscanning. *N. En. J. Med.*, **298**, 1384.

GOLDENBERG, D.M., SHARKEY, R.M. & PRIMUS, F.J. (1978*b*). Immunocytochemical detection of carcinoembryonic antigen in conventional histopathology specimens. *Cancer,* **42,** 1546.

JONES, B.G. (for the British Stomach Cancer Group). (1981). Design and progress of a multi-centre trial of adjuvant chemotherapy in operable gastric cancer. In: *Progress and Perspectives in the Treatment of Gastro-intestinal Tumours.* Pergamon Press, p. 36.

LEE, P.K., MORI, T., SHIMANO, T., MASUZANA, M. & KOSAKI, G. (1978). Immunohistological studies of CEA, AFP and CPALP in Gastric Cancer. *Scand. J. Immunol.,* **8,** (Suppl. 8) 485.

N.I.H. CONCENSUS STATEMENT. (1981). Carcinoembryonic antigen. Its role as a marker in the management of cancer. *Br. Med. J.,* **282,** 373.

PRIMUS, F.J., KUHNS, N.J. & GOLDENBERG, D.M. (1983). Immunological heterogeneity of carcinoembryonic antigen: Immunohistochemical detection of carcinoembryonic antigen determinants in colonic tumours with monoclonal antibodies. *Cancer Res.,* **43,** 693.

ROGERS, G.T., RAWLINS, G.A., KEEP, P.A., COOPER, E.H. & BAGSHAWE, K.D. (2981). Application of monoclonal antibodies to purified CEA in chemical radioimmunoassay of human serum. *Br. J. Cancer,* **44,** 371.

ROWLAND, G.F., SIMMONDS, R.G., CORVALAN, J.R.F. & 9 others. (1982). The potential use of monoclonal antibodies in drug targeting. *Prot. Biol. Fluids,* **29,** 921.

SKINNER, J.M. & WHITEHEAD, R. (1982). Tumour markers in carcinoma and premalignant states of the stomach in humans. *Eur. J. Clin. Oncol.,* **18,** 227.

WAGENER, C., MULLER WALLRAF, R., NISSON, S., GRONER, J., & BREUER, H. (1981). Localization and concentration of carcinoembryonic antigen (CEA) in gastrointestinal tumours: correlation with CEA levels in plasma. *J. Nat Cancer Inst.,* **67,** 539.

WOODHOUSE, C.S. (1982). *An Investigation of Human Lung Tumour Antigens.* Ph.D. Thesis Univ. of Birmingham.